# Sea Cucumber (*Stichopus japonicas*) F2 Enhanced TRAIL-Induced Apoptosis via XIAP Ubiquitination and ER Stress in Colorectal Cancer Cells

**DOI:** 10.3390/nu11051061

**Published:** 2019-05-11

**Authors:** Jung Lim Kim, Seong Hye Park, Soyeon Jeong, Bo Ram Kim, Yoo Jin Na, Min Jee Jo, Yoon A Jeong, Hye Kyeong Yun, Dae Yeong Kim, Bu Gyeom Kim, SangGuan You, Sang Cheul Oh, Dae-Hee Lee

**Affiliations:** 1 Department of Oncology, Korea University Guro Hospital, Seoul 152-703, Korea; clickkjl@naver.com (J.L.K.); limlab7@gmail.com (S.J.); ilovewish777@naver.com (B.R.K.); 2 Graduate School of Medicine, Korea University College of Medicine, Seoul 152-703, Korea; psh3938@hanmail.net (S.H.P.); wing1278@naver.com (Y.J.N.); minjeeyoyo@nate.com (M.J.J.); leomi2614@naver.com (Y.A.J.); katecoco@hanmail.net (H.K.Y.); derrickdyblue22@gmial.com (D.Y.K.); qnrua10047@naver.com (B.G.K.); 3 Department of Marine Food Science and Technology, Gangneung-Wonju National University, Gangwon 210-702, Korea; sangguanyou@gmail.com

**Keywords:** sea cucumber (SC), endoplasmic reticulum (ER) stress, X-linked inhibitor of apoptosis protein (XIAP), c-Jun N-terminal kinase (JNK)

## Abstract

Natural products have shown great promise in sensitizing cells to TNF-related apoptosis-inducing ligand (TRAIL) therapy. Sea cucumber (SC) extracts possess antitumor activity, and hence their potential to sensitize colorectal cancer (CRC) cells to TRAIL therapy was evaluated. This study used Western blotting to evaluate the combination effects of SC and TRAIL in CRC, and determined the molecular mechanism underlying these effects. SC fractions and TRAIL alone did not affect apoptosis; however, combined treatment dramatically induced the apoptosis of CRC cells, but not of normal colon cells. Combined treatment induced the expression of apoptotic proteins (poly (ADP-ribose) polymerase (PARP), caspase 3, and 8), and this effect was markedly inhibited by the ubiquitination of X-linked inhibitor of apoptosis protein (XIAP). SC did not affect the mRNA levels, but it increased proteasomal degradation and ubiquitination of the XIAP protein. Furthermore, SC induced reactive oxygen species (ROS) production, thereby activating c-Jun N-terminal kinase (JNK) and endoplasmic reticulum (ER) stress-related apoptotic pathways in CRC. Altogether, our results demonstrate that the SC F2 fraction may sensitize CRC cells to TRAIL-induced apoptosis through XIAP ubiquitination and ER stress.

## 1. Introduction

Colorectal cancer (CRC) is known as the third most common cancer in the world, with an estimated 1.4 million newly diagnosed cases in 2012 [1]. Treatment of CRC commonly involves a combination of three classic strategies of oncology: chemotherapy, surgery, and radiation. The approach of using chemotherapy drugs for CRC has limitations such as the acquisition of resistance to drugs after repeated administration. Many chemotherapy regimens have been clinically investigated for CRC treatment; however, novel therapeutic agents for CRC therapy are still needed. TNF-related apoptosis-inducing ligand (TRAIL) is one of the members of the tumor necrosis factor (TNF) family of death ligands that induce programmed cell death via interaction with its corresponding death receptors (DRs). TRAIL selectively induces apoptosis in a variety of tumor cells but shows little or no toxicity to normal cells [2]. TRAIL interacts mainly with two distinct receptors, namely, TRAIL receptors 1 (DR4) and 2 (DR5), and recruits Fas-associated death domain protein (FADD) and procaspase-8/10 to form the death-inducing signaling complex (DISC) [3]. TRAIL is a potential agent for cancer treatment, with its agonists being evaluated in phases I and II of clinical trials [4,5]. However, TRAIL therapy has been limited due to the resistance to TRAIL-induced apoptosis [6]. Therefore, understanding the underlying molecular mechanism(s) for TRAIL-induced resistance will help to identify sensitizing targets of cell death. TRAIL-resistant cancer cells can be sensitized by anti-cancer agents, thereby recommending a possibility of combination therapy.

The endoplasmic reticulum (ER) plays an important role in protein synthesis and maturation, protein folding, calcium homeostasis and lipid synthesis [7]. Moderate ER stress serves as a protective mechanism for cell proliferation, but severe or long-term ER stress may lead to initiation of apoptosis [8,9]. Recent studies indicate that reactive oxygen species (ROS), anti-cancer agents, hypoxia, and radiation aggravate ER-stress response and activate the ER stress-mediated apoptosis pathway in cancer [10,11,12,13]. Therefore, we infer that the sea cucumber fraction may activate the ER stress-related apoptosis via exacerbating ROS generation and causing ER-stress response.

Sea cucumber (SC) is an edible marine invertebrate belonging to the classes Holothuroidea and Phylum Echinodermata. SC is rich in biological compounds, including collagen protein, vitamins, triterpene glycosides, saponins and polysaccharides. It has been proposed that SC exhibits preventive or curative effects against various diseases. The methanolic, ethanolic, and aqueous extracts of SC are known to possess antioxidant, antibiotic, anti-inflammatory and anticancer activities [14,15]. The previous study reported that compounds extracted from SC acted as MDM2 and CXCR4 inhibitors, which inhibited the growth of cancer cells [16]. Recently, the water-soluble protein-sulfated fucan (PSF) complex was isolated from the *Stichopus japonicas* and four refined fractions (F1, F2, F3, and F4) were obtained using anion exchange chromatography. SC F1 was eluted with distilled water, F2 was eluted with 0.5 M NaCl, F3 was eluted with 1.0 M NaCl, and F4 was eluted with 1.5 M NaCl. F1 and F2 are quite similar, mostly composed of neutral sugars and proteins with small amounts of sulfates and no uronic acid, whereas F3 and F4 are mostly composed of fucose with limited amounts of galactose, glucose and mannose [17].

In the present study, we verify a novel antitumor mechanism of the SC fraction. The SC fraction, an extract of *S. japonicas*, enhanced the sensitivity to TRAIL-induced apoptosis in CRC cells through decreasing the expression of X-linked inhibitor of apoptosis protein (XIAP) and increasing the ER-stress.

## 2. Materials and Methods

### 2.1. Cell Culture

The human CRC (DLD-1, colo205, HCT116, HT29 and SW620) and normal colon cell lines (CCD18Co) were purchased from the American Type Culture Collection (ATCC, Manassas, VA, USA) and maintained according to the ATCC’s instructions. The human normal colon cell line (CCD18Co) was maintained in growth medium (EMEM), whereas all the other cell lines were cultured in RPMI 1640 supplemented with 10% Fetal Bovine Serum (FBS) and l-glutamine and grown in a humidified 5% CO_2_ incubator at 37 °C.

### 2.2. Reagents and Antibodies

SC F2 was kindly provided by Gangneung-Wonju National University [17]. Drug treatments were performed by inhaling the medium and replacing it with drugs containing medium. TRAIL protein (recombinant human) was purchased from Millipore (Millipore, Darmstadt, Germany). Protein G PLUS-agarose, anti-Bcl-2, anti-Bax, anti-Ub, anti-SOD 3, anti-DR5, anti-DR4 and anti-Bcl-xL were purchased from Santa Cruz Biotechnology (Santa Cruz, CA, USA). Anti-Mcl-1, anti-XIAP, anti-cleaved caspase-9, anti-cleaved caspase-8, anti-cleaved caspase-3, anti-Noxa, anti-Puma, anti-Bim, anti-Survivin, anti-p-JNK, anti-JNK, anti-CHOP, anti-PERK, anti-p-PERK, anti-ATF6, anti-IRE-1α, anti-ATF4, anti-eIF2a, anti-p-eIF2a, anti-GRP78, anti-GRP94, anti-SOD 1, anti-SOD 2 and anti-PARP-1 were purchased from Cell Signaling Technology (Beverly, MA, USA). Anti-p-IRE-1α and anti-catalase were purchased from Abcam (Cambridge, England). Anti-actin, DCFH-DA, N-acetyl L-cysteine (NAC) and H_2_O_2_ were purchased from Sigma (Sigma, St. Louis, MO, USA).

### 2.3. Survival Assay

DLD-1 cells were seeded in tissue culture-coated 96-well plates and treated as described in the results. DLD-1 cells were then incubated with 3-(4,5-dimethylthiazol-2-ly)-2,5-diphenyl tetrazolium bromide (MTT) (Roche Molecular Biochemical, Indianapolis, IN, USA) for 3 h at 37 °C. Absorbance at 450 nm was recorded using an enzyme-linked immunosorbent assay (ELISA) microplate reader.

### 2.4. Transfection of Small Interfering RNA (siRNA)

XIAP siRNA (Cat. No. SC-37508) and negative Control siRNA (Cat. No. SC-37007) were purchased from Santa Cruz Biotechnology (Santa Cruz, CA, USA). C/EBP-homologous protein (CHOP) siRNA was obtained from Bioneer. DLD-1 cells were transfected with siRNA (200 nM) oligonucleotides using Lipofectamine RNAi Max (Invitrogen, Carlsbad, CA, USA) according to the manufacturer’s instructions. 24 h post-transfection, the DLD-1 cells (3.5 × 10^5^) were treated with TRAIL or SC F2 for further analysis. The transfection efficiency was confirmed by analyzing the expression levels of XIAP and CHOP using Western Blot at 24 h post-transfection, and then determined by Western Blot quantification with ImageJ of Hossein Davarinejad.

### 2.5. Transient Transfection

For transient overexpression experiments, DLD-1 cells were transfected either with 0.5 μg empty vector (pcDNA3) or 0.5 μg plasmid encoding Myc-tagged XIAP cDNA (Plasmid #11833 from addgene) using Lipofectamine 2000 (Invitrogen, USA) according to the manufacturer’s instructions. The transfection efficiency was confirmed by analyzing the expression levels of XIAP using Western Blot at 24 h post-transfection, and then determined by Western Blot quantification with ImageJ of Hossein Davarinejad.

### 2.6. Western Blotting

Western blotting was determined as previously described [18].

### 2.7. Apoptosis Assay

Apoptotic cells were stained using an annexin V-fluorescein isothiocyanate (FITC) kit (BioBud, Cat LS-02-100), according to the manufacturer’s instructions, and were then analyzed. Apoptosis in the DLD-1 cells was immediately analyzed by flow cytometry (Beckman Coulter, Brea, CA, USA).

### 2.8. Co-Immunoprecipitation

Co-immunoprecipitation was determined as previously described [18].

### 2.9. Colony Formation Assay

DLD-1 cells were seeded into a 6-well plate at a density of 500 cells/well and incubated at 37 °C. Colony formation on the plate was conducted in 6-well cell culture plates as previously described. The medium was changed every three days. After two weeks, the DLD-1 cells were washed with phosphate-buffered saline (PBS), fixed with 4% paraformaldehyde for 30 min, followed by staining with crystal violet for 30 min. Colonies were then visualized and counted.

### 2.10. Reverse Transcriptase Polymerase Chain Reaction

Total RNA extraction was performed using the TRIzol reagent (Life Technologies, Rockville, MD, USA), according to the manufacturer’s instructions. Total RNA was reverse transcribed to obtain cDNA, which was amplified using an RT-PCR kit (Life Technologies) and the following XIAP primers: forward, 5′-GTGCCACGCAGTCTACAAATTCTGG-3′, reverse, 5′-CGTGCTTCATAATCTGCCATGGATGG-3′ [19], and β-actin primers: forward, 5′-ACC CAG ATC ATG TTT GAG AC-3′, reverse, 5′-GGA GTT GAA GGT AGT TTC GT-3′.

### 2.11. Real-Time PCR

Total RNA was extracted using the TRIzol reagent (Life Technologies). Amplification of the transcripts was performed by reverse transcriptase polymerase chain reaction kit (Life Technologies, Carlsbad, CA, USA). The PCR was performed on Applied Biosystems 9700 RT-PCR using gene-specific oligonucleotide primers and Taqman probes (Applied Biosystems, Foster City, CA, USA). The probes were as follows: Glyceraldehyde-3-Phophate Dehydrogenase (GAPDH) (Hs99999905_m1), XIAP (Hs00745222_s1). For mRNA quantification, gene expression was normalized by GAPDH. Relative expression levels were calculated using the 2(-Delta Delta C(T) method [20].

### 2.12. ROS Generation

ROS generation was assessed in DLD-1 cells treated with SC F2 for 30 or 60 min. Intracellular ROS levels were determined using 2′,7′-dichlorodihydrofluorescein diacetate (DCFH-DA), as previously described [21].

### 2.13. Tumor Xenograft Experiment

All animal experiments were carried out in accordance with animal care guidelines approved by the Korea University Institutional Animal Care and Use Committee (IACUC, KOREA-2018-0167). We have received a Certificate of IACUC Approval. Four-week-old female BALB/c nude mice (n = 20; weight, 18–23 g) were acquired from Orient Bio (Korea) and housed in a specific, pathogen-free environment. The animals were acclimated for one week prior to the study and were provided with unlimited access to food and water. DLD-1 cells (5 × 10^6^) in 100 µL of culture medium were mixed with 20 µL of Matrigel and implanted subcutaneously into the right flank of five-week-old BALB/c nude female mice. Tumor size was determined by measuring the diameter (width, length) of the tumor every two days with a digital caliper. Mice sacrifice was carried out by CO_2_ asphyxiation. Mice from all groups were sacrificed at day 19. Mouse sacrifice was performed by waiting 2 min after 1 min of CO_2_ injection in the CO_2_ chamber. The CO_2_ displacement rate was limited to 10% to 30% of the cage volume per minute.

### 2.14. Statistical Analysis

Statistical analysis was performed using GraphPad InStat 5 software (GraphPad Software, Inc., San Diego, CA, USA). Values are expressed as mean ± SD. Data were compared using two-tailed Student’s t-test or one-way analysis of variance and Tukey’s post hoc test. *p* < 0.05 was considered to indicate a statistically significant difference.

## 3. Results

### 3.1. SC F2 Enhances TRAIL-Induced Apoptosis in CRC Cells

**Figure 1:**
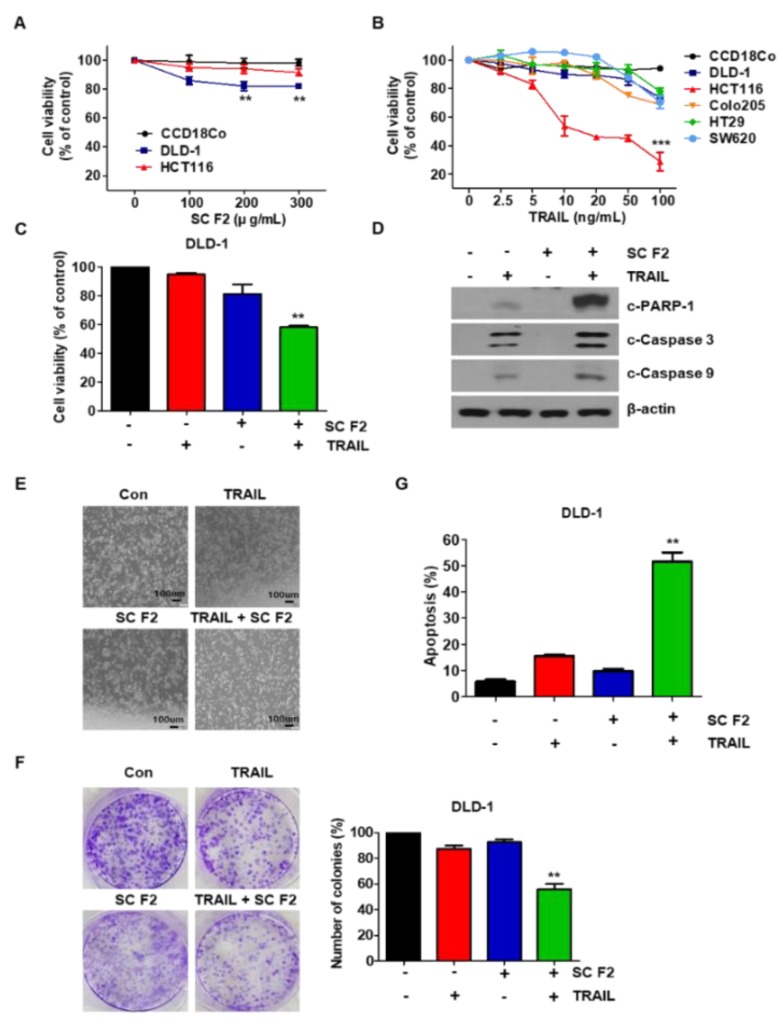
**Sea cucumber** (SC) F2 significantly activated TNF-related apoptosis-inducing ligand (TRAIL)-induced apoptosis of human colorectal cancer (CRC) cell lines. (**A**) Cytotoxicity of SC F2 (**B**) Cytotoxicity of TRAIL (**C**) Cytotoxicity of either substance alone, or the combination of SC F2 and TRAIL in DLD-1 cells (mean ± SD, n = 5) was evaluated using MTT (3-(4,5-dimethylthiazol-2-ly)-2,5-diphenyl tetrazolium bromide) assay. Cells were treated with Dimethyla sulfoxide (DMSO) (mock control) or SC F2 at various concentrations (0–300 μg/mL) for 20 h. Cells were treated with TRAIL at various concentrations (0–100 ng/mL) for 4 h. To check the cytotoxic effect of the combination of SC F2 and TRAIL in DLD-1 cells, they were cultured in the presence or absence of TRAIL (20 ng/mL) and/or SC F2 (200 μg/mL) for 24 h. (**D**) Western blotting of cleaved PARP-1, cleaved caspase-9 and cleaved caspase-3 antibodies. (**E**) DLD-1 cells were treated with SC F2 or TRAIL alone or SC F2 in combination with TRAIL for 24 h. Cell morphology was observed using the optical microscope. Scale bar: 100 μm. (**F**) DLD-1 cells plated in 6-well cell culture plates were treated with 200 μg/mL SC F2, 20 ng/mL TRAIL, or their combination. After 12 days, the plates were stained with crystal violet dye, and colonies were visualized and counted using a digital camera. Quantitative analysis of data. (**G**) DLD-1 cells were stained with annexin V and propidium iodide (PI), for flow cytometry analysis. Percentage of apoptosis in cells treated with SC F2 or TRAIL alone or in combination. Experiments were performed at least three times. Error bars represent standard error of the mean (SEM) from three independent experiments. ** *p* < 0.05, *** *p* < 0.01.

We found that the combination of SC F2 and TRAIL among the four extracts obtained from SC (F1, F2, F3, F4) was the most effective in CRC cells (data not shown). Thus, the SC F2 fraction was used for further experiments. We examined the cytotoxicity of SC F2 in CRC cancer cell lines. All the tested cancer cell lines showed dose-dependent SC F2-induced apoptosis, whereas normal primary colon cells (CCD18Co) were drug-resistant (Figure 1A). Additionally, we also observed that TRAIL alone inhibited cell proliferation in all the tested cell lines (Figure 1B). The combined effect of SC F2 and TRAIL on cell viability in the indicated CRC cell lines was investigated. Cytotoxicity was significantly enhanced by the combined treatment of SC F2 and TRAIL in DLD-1 cells (Figure 1C). In these cells, SC F2 enhanced TRAIL-induced activation of caspase-9 and caspase-3, leading to increased PARP cleavage (Figure 1D). We found the same results in HCT116, another CRC cell line (data not shown). Activation of apoptosis by SC F2 in combination with TRAIL in DLD-1 cells was further examined by observing cell morphological changes under a light microscope. Figure 1E shows that SC F2 and TRAIL co-treated cells displayed more apoptotic morphology than cells treated with either substance alone (Figure 1E). We have confirmed similar results in HCT116, another CRC cell line (data not shown). Next, we examined the long-term effect of the SC F2 and TRAIL combination on clonogenic survival and observed that this combination proved to be effective in preventing colony formation (Figure 1F). As shown in Figure 1G, we found that SC F2 increased TRAIL-induced apoptosis in DLD-1 cells. These results indicate that SC F2 potentiates TRAIL-induced apoptosis.

### 3.2. SC F2 Sensitizes CRC Cells to TRAIL-Induced Apoptosis Via XIAP

**Figure 2:**
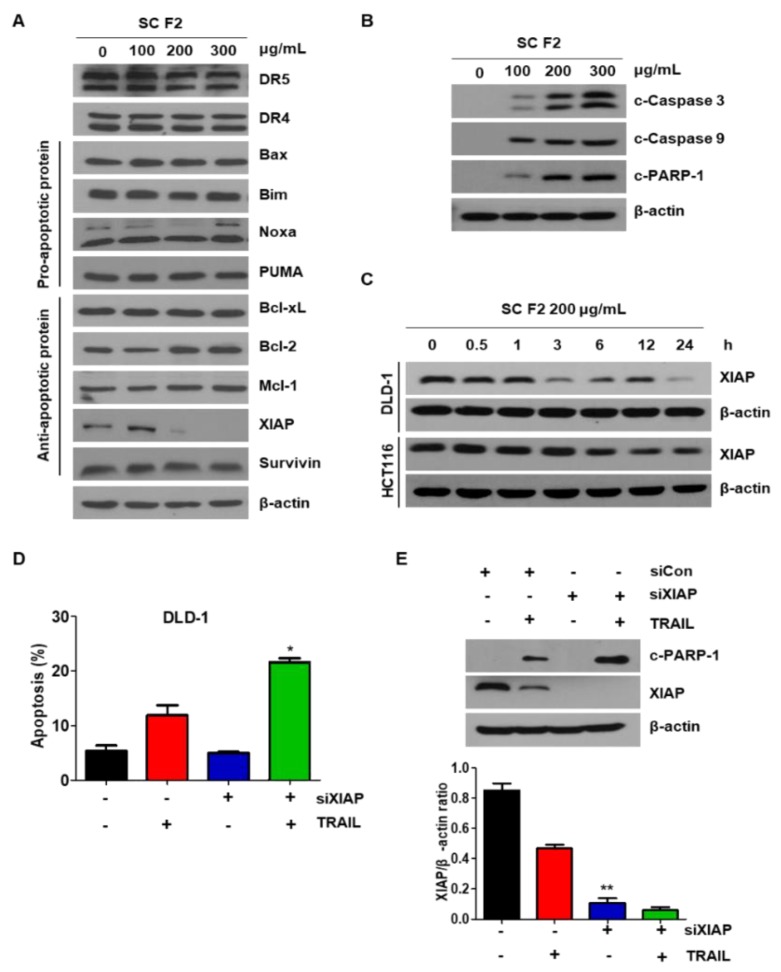
Suppression of XIAP by SC F2 sensitizes cells to TRAIL-mediated apoptosis. (**A**) DLD-1 cells were treated with the indicated concentrations of SC F2 for 20 h. Western blotting of death receptor (DR4, DR5), anti-apoptotic and pro-apoptotic protein. (**B**) DLD-1 cells were treated with indicated SC F2 doses for 20 h. Cell lysates were analyzed by Western blotting using anti-caspase 9, anti-caspase 3, and anti-cleaved PARP-1 antibodies. (**C**) DLD-1 and HCT116 cells were exposed to 200 µg/mL of SC F2 for the indicated time intervals. Cell lysates were analyzed by Western blotting using an anti-XIAP antibody. (**D**) XIAP was silenced by XIAP siRNA in DLD-1 cells. The cells were then treated with TRAIL for 4 h followed by flow cytometry analysis. Error bars represent standard error of the mean (SEM) from three separate experiments. “*” represents a statistically significant difference between TRAIL + siCon-treated and TRAIL + siXIAP-treated cells at *p* < 0.05. (**E**) XIAP was silenced by XIAP siRNA in DLD-1 cells. The cells were then treated with TRAIL for 4 h followed by Western blotting. ** *p* < 0.005.

To investigate the mechanism of SC F2 to enhance TRAIL-induced apoptosis, expression levels of death receptors, pro-apoptotic, and antiapoptotic proteins were checked. The protein levels of death receptors (DR4 and DR5) as well as members of the caspase inhibitor protein family remained unchanged upon SC F2 treatment in DLD-1. However, a decrease in the level of XIAP was observed in DLD-1 (Figure 2A). The same results were confirmed in other CRC cells (data not shown). Levels of apoptotic proteins (c-PARP1, c-caspase 3, and c-caspase 9) were promoted in a dose-dependent manner in SC F2-treated cells (Figure 2B), confirming the induction of apoptosis. Further, we observed that the level of XIAP was decreased by SC F2 in a time-dependent manner in DLD-1 and HCT116 cell lines (Figure 2C). Finally, the knockdown of XIAP by siRNA promoted TRAIL-induced apoptosis (Figure 2D,E), inferring that the sensitizing effect of SC F2 occurs through down-regulation of XIAP level in CRC cells.

### 3.3. SC F2-Induced TRAIL Apoptosis of CRC Is Mediated by ER Stress and JNK Phosphorylation

**Figure 3:**
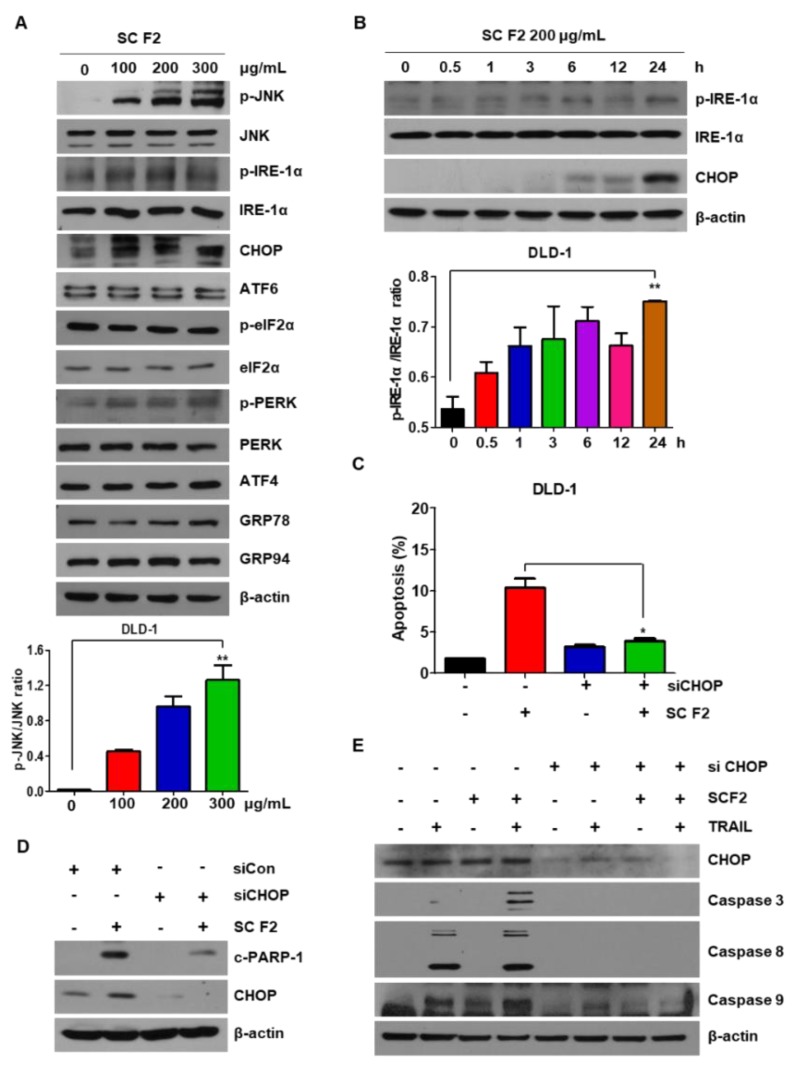
SC F2-induced C/EBP-homologous protein (CHOP) up-regulation is mediated through selective induction of IRE1 α-JNK endoplasmic reticulum (ER) stress signaling. DLD-1 cells were treated with indicated concentrations of SC F2 for 20 h (**A**), or treated with SC F2 (200 μg/mL) for indicated time points (**B**). The protein expression levels of the ER stress pathway were measured by immunoblotting using corresponding antibodies. (**C**) DLD-1 cells were transfected with siRNA against CHOP or control. After 24 h, cells were treated with SC F2 (200 μg/mL) for 20 h followed by flow cytometry analysis. Error bars represent standard error of the mean (SEM) from three separate experiments. (**D**) CHOP was silenced by CHOP siRNA in DLD-1 cells. The cells were then treated with SC F2 (200 μg/mL) for 20 h followed by Western blotting using an anti-cleaved PARP-1 antibody. (**E**) DLD-1 cells were transiently transfected with CHOP siRNA (siCHOP). Twenty-four hours after transfection, cells were pretreated with or without SC F2 (200 μg/mL) for 20 h and then TRAIL (20 ng/mL) for 4 h. “*” or “**” represents a statistically significant difference between untreated control cells and drug treated cells at *p* < 0.05 or *p* < 0.01, respectively.

Recent studies indicate that ER stress plays an important role in the regulation of apoptosis [9]. To confirm whether ER stress plays a role in SC F2-mediated apoptosis, we investigated the expression of ER stress-related proteins in DLD-1 cells. Of note, SC F2 showed dose- and time-dependent activation of these proteins (Figure 3A,B), indicating that SC F2-induced cell apoptosis is also mediated by the ER stress signaling pathway. Next, we used siRNA to silence CHOP expression and found that SC F2-induced CHOP and PARP levels decreased dramatically (Figure 3C,D). In Figure 3E, the silencing of CHOP decreased SC F2-induced TRAIL sensitivity. Our results suggest that activated ER stress was involved in SC-mediated apoptosis.

### 3.4. ROS Generation Mediates SC F2-Activated ER Stress Pathways

**Figure 4:**
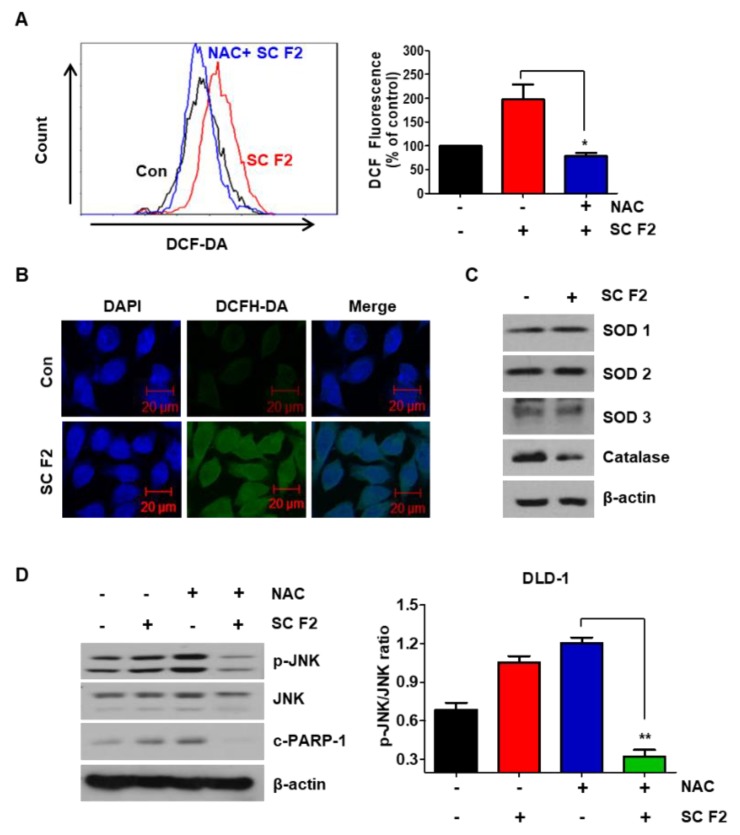
SC F2-induced ROS generation is mediated through selective decrease of catalase. (**A**) ROS production was assessed by DCFH-DA, following 1 h pre-treatment with 200 μg/mL of SC F2 in DLD-1 cells. (**B**) DLD-1 cells were treated with SC F2 (200 μg/mL) for 20 h, cells were stained with FITC probe DCFH-DA (10 μM) for 30 min at 37 °C, and visualized under a confocal microscope. (**C**) DLD-1 cells were treated with SC F2 (200 μg/mL) for 20 h. The protein expression levels of ROS-related proteins were measured by immunoblotting using corresponding antibodies. (**D**) DLD-1 cells were pre-treated with 10 mM *N*-acetyl-l-cysteine (NAC) for 1 h, followed by treatment with or without SC F2 (200 μg/mL). Cells were then lysed and subjected to Western blotting. “*” or “**” represents a statistically significant difference between untreated control cells and drug treated cells at *p* < 0.05 or *p* < 0.01, respectively.

To investigate the potential role of ROS in SC F2-induced apoptosis, we measured the levels of ROS in SC F2-treated CRC cells using DCFH-DA dye. Figure 4A,B show that significant fluorescence signals were detected in cells exposed to SC F2. Furthermore, treatment of the ROS scavenger NAC inhibited the SC F2-induced ROS accumulation. We also observed that the level of catalase, an enzyme responsible for hydrogen peroxide degradation, decreased in SC F2-treated cells (Figure 4C). As shown in Figure 4D, the SC F2-induced PARP level in CRC cells was effectively blocked on co-culturing with NAC. In addition, cells were pretreated with NAC to prevent the down-regulation of SC F2-induced p-JNK expression by blocking the production of ROS.

### 3.5. SC F2 Induces XIAP Degradation through the Ubiquitin Signaling Pathway

**Figure 5:**
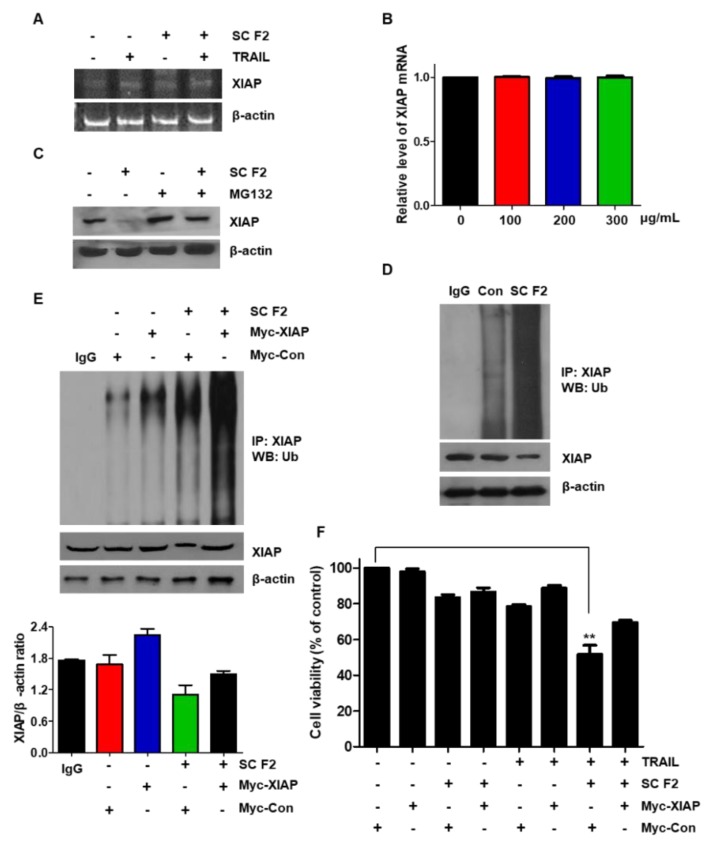
XIAP plays a role in the sensitization function of SC F2. DLD-1 cells were treated with SC F2 at indicated doses for 20 h. The mRNA expression levels of XIAP and actin were measured by reverse transcriptase-PCR (**A**) and real time-PCR (**B**). (**C**) SC F2-treated DLD-1 cells were treated in the presence or absence of MG132 and Western blot analysis using anti-XIAP and anti-actin antibodies was carried out. (**D**) SC F2-treated DLD-1 cells were subjected to immunoprecipitation with an anti-XIAP antibody and immunoblotted for anti-Ub. (**E**) Empty vector (0.5 μg) or XIAP vector (0.5 μg) transiently over-expressing. DLD-1 cells were treated with 200 μg/mL SC F2 for 20 h and immunoprecipitated with the anti-XIAP antibody or with IgG, and immunoblotted for Ub. Western blot analysis showing XIAP and actin. (**F**) Measurement of apoptotic index in Myc-control and Myc-XIAP-treated DLD-1 cells by MTT assay after SC F2 and TRAIL treatment. Error bars represent standard error of the mean (SEM) from three separate experiments. “**” represents a statistically significant difference between TRAIL treated on empty vector transfected cells and TRAIL treated on Myc-XIAP vector transfected cells at *p* < 0.01.

We observed that SC F2 downregulated XIAP protein expression, but it did not affect XIAP mRNA at transcriptional level (Figure 5A,B). We also observed the effect of the proteasome inhibitor (MG132) on SC F2-induced XIAP degradation. MG132 prevented SC F2-induced down-regulation of XIAP (Figure 5C,D). These results suggest that SC F2 decreases XIAP levels through activation of the ubiquitin-proteasome-dependent pathways. Next, we transiently overexpressed XIAP by treating DLD-1 cells either with Myc-control or Myc-XIAP and/or SC F2. Overexpression of the XIAP vector did not significantly increase the XIAP protein level, only by about 1.4-fold, and we confirmed that ubiquitination of XIAP was increased in SC F2-treated DLD-1 cells by immunoprecipitating XIAP and blotting for Ubiquitin (Figure 5E). The cell death indication was then analyzed with the MTT assay. We found that the cell death indication of these cells significantly decreased through overexpression of XIAP level by Myc-DNA treatment (Figure 5F). These studies suggest that SC F2-induced XIAP degradation is mainly ubiquitin-dependent.

### 3.6. SC F2 Increases TRAIL-Induced Apoptosis In Vivo

**Figure 6:**
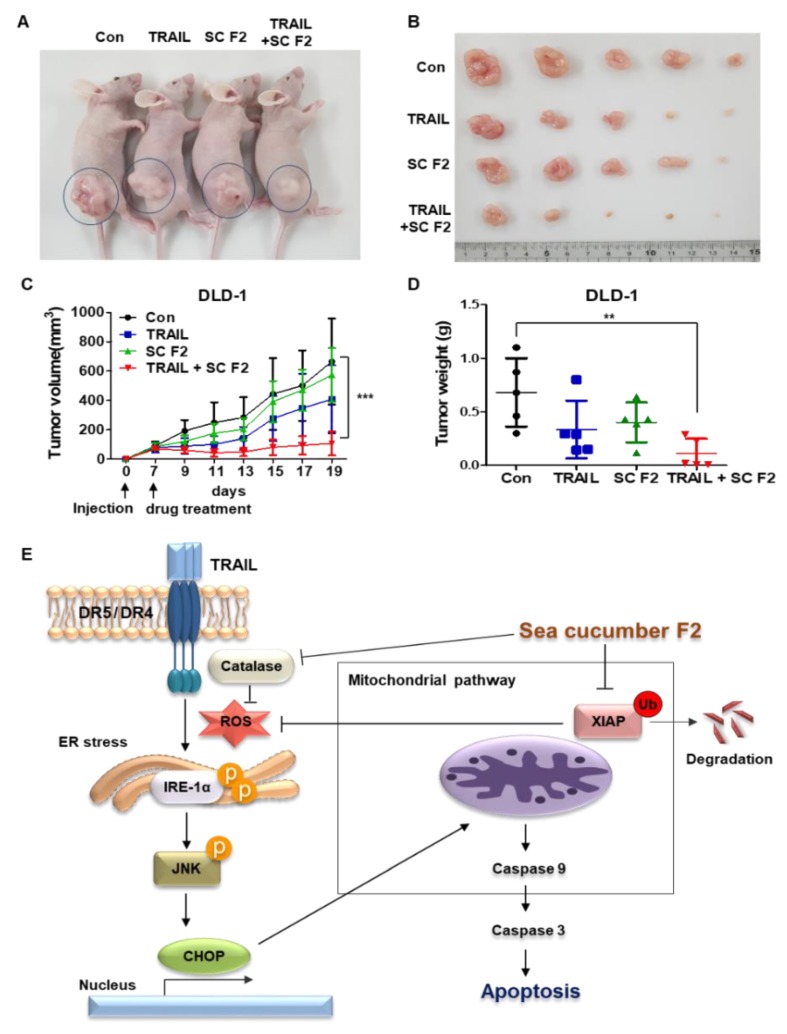
SC F2 enhanced TRAIL-induced apoptosis in vivo. (**A–C**) DLD-1 cells were inoculated into nude mice (n = 5 per group) at 5 × 10^6^ per mouse subcutaneously. Mice received 4 ng/kg TRAIL and 50 mg/kg SC F2 either alone or in combination at day 19 after tumor implantation. Representative tumors of each group are shown. Tumor volume was calculated every 2 days for 19 days according to the following equation: tumor volume (mm^3^) = π/6 × length × (width) ^2^. Maximum tumor area and its corresponding section were calculated using MetaMorph software (Molecular Devices). (**D**) The average tumor weight in nude mice after administration of TRAIL and SC F2 alone or in combination. (**E**) Schematic diagram for a working model of SC F2 sensitizing TRAIL-induced apoptosis. *** *p* < 0.001.

DLD-1 cells (5 × 10^6^) were subcutaneously injected into BALB/c nude mice. We randomly divided the mice into four groups and treated them with TRAIL (4 ng/kg) and/or SC F2 (50 mg/kg) three times per week. The combination of TRAIL and SC F2 significantly decreased tumor growth compared to that of the control or the single-treatment groups (Figure 6A–D). The diameter of the largest subcutaneous tumor observed in the study for each group is as follows; control group 20.39 mm, TRAIL group 16.51 mm, SC F2 group 16.95 mm and TRAIL + SC F2 group 8.99 mm. The mean tumor weights were 0.682 g for the control group, 0.336 g for the TRAIL group, 0.402 g for the SC F2 group and 0.112 g for the TRAIL + SC F2 group. The maximum tumor burden (percentage of the animal’s body weight) observed in the study for each group is as follows; control group 5.5%, TRAIL group 4.0%, SC F2 group 3.2% and TRAIL + SC F2 group 1.4%. SC F2 and TRAIL did not cause a significant impact on the body weight of the mouse (data not shown). We also confirmed that the tumor had not spread to other parts of the body at the primary position of the mice. We confirmed that TRAIL induces apoptosis in CRC cells in combination with SC F2. Figure 6E shows that SC F2 enhances TRAIL-induced apoptosis through ER stress.

## 4. Discussion

TRAIL possesses great potential to create a new generation of anti-tumor agents. However, some tumors remain resistant to TRAIL-mediated cell death by activating apoptosis-related proteins such as death receptors (DR4 and DR5) and anti-apoptotic proteins (c-FLIP, IAPs and Mcl-1) [6,22,23]. Therefore, a strategy to enhance TRAIL sensitivity is required.

In the present study, a new compound of natural origin, SC fraction, activated ER stress through XIAP ubiquitination and enhanced sensitivity to TRAIL-induced apoptosis in CRC cells. Combined treatment leads to the down-regulation of the anti-apoptotic XIAP protein. Normal colon (CCD-18Co) cells were unaffected by the combined treatment (data not shown), whereas apoptotic cell death was induced in the CRC cell line (DLD-1). These data suggest that the natural compound SC fraction could be an effective TRAIL sensitizer, and combination therapy of SC fraction with TRAIL may be an effective therapeutic strategy for CRC.

Many studies have reported that sea cucumber extract contains several compounds, including mono sulfated tritepenoid glycoside Frondoside A, disulfated glycoside Frondoside B, trisulfated glycoside Frondoside C, eicosapentaenoic acid, fucosylated chondroitin sulfate, nobiliside D, 12-methyltetradecanoic acid, sphingoid and canthaxanthin/astaxanthin, that have induced apoptosis in solid cancers, including breast, liver, renal, cervical, lung, and colorectal cancer cells [15,24,25,26]. Amidi et al. reported that (Z)-2,3-diphenylacrylonitrile molecules isolated from Persian Gulf SC were identified as potential anti-cancer agents [27]. In our study, the crude and fractionated sulfated fucan from the sea cucumber, *Stichopus japonicas*, was used to investigate its anti-tumor activity [17]. You et al. reported that sulfated fucan enhanced Natural Killer (NK) cell cytotoxicity against HeLa, HepG2, and HT-29 cells [28]. Another component of sea cucumber, Frondoside A, was able to potentiate the anti-cancer effects of gemcitabine, paclitaxel, cisplatin, 5-Fluorouracil, and oxaliplatin in the treatment of pancreatic, breast, lung, and colorectal cancers [29,30,31,32]. Although studies on the combination of components extracted from sea cucumber and cytotoxic agents have been reported, no studies on the combination of sulfated fucan have been conducted.

Suppression of XIAP may represent a key anti-apoptotic mechanism in cancer cells. It aggravates the prognosis and recurrence of cancer [33,34]. Inhibition of XIAP by a variety of chemical compounds has been reported. A recent study indicated that XIAP inhibitors synergize with TRAIL to induce apoptosis and suppress various tumor growths. [34,35] For example, flavopiridol and chetomin enhance TRAIL-induced apoptosis via degradation of XIAP [36,37]. Additionally, small molecule XIAP inhibitors sensitized pancreatic cancer cells to TRAIL-induced apoptosis via the decrease of XIAP [35]. In our study, one mechanism of SC-mediated TRAIL sensitization was the down-regulation of XIAP expression. To our knowledge, this is the first report describing the apoptotic mechanism of the combination of SC and TRAIL in colorectal cancer cells. These results suggest the rationale for further pre-clinical development of XIAP inhibitors and TRAIL against colorectal cancer.

Hiramatsu et al. reported that the down-regulation of XIAP is a conserved consequence of ER stress and that this drop correlates with the emergence of apoptosis [38]. We therefore hypothesized that the induction of apoptosis in CRC cells by the drug combination might be related to ER stress activation. ER stress is involved in cell survival and tumor progression [9,39]. It is also an alternative pathway to the mitochondrial pathway for ROS-regulated apoptosis. The accumulation of unfolded response protein (UPR) occurs through ER transmembrane proteins ATF6, IRE1α, and PERK, which carry the response downstream to induce transcription of C/EBP homologous protein (CHOP) [40]. In the present study, multiple ER stress markers such as phospho-IRE1α, phospho-PERK, and CHOP were up-regulated upon treatment with SC F2. These findings demonstrated that SC F2 enhanced TRAIL-induced apoptosis via ER stress. RNAi-mediated silencing of XIAP or CHOP in DLD-1 cells suppressed drug combination-induced apoptosis, further confirming the role of XIAP and ER stress in SC F2-mediated sensitization to TRAIL-induced apoptosis.

## 5. Conclusions

In summary, we showed that the pretreatment of CRC cells with SC F2 resulted in a significant potentiation of TRAIL-induced apoptosis via XIAP degradation and induction of the ER stress pathway. Our findings suggest a rationale for the potential application of the combination of SC and TRAIL in CRC therapy.

## Abbreviations

TRAIL	TNF-related apoptosis-inducing ligand
XIAP	X-linked inhibitor of apoptosis protein
CHOP	C/EBP-homologous protein
eIF2α	eukaryotic translation initiation factor 2α
ER	endoplasmic reticulum
FITC	fluorescein isothiocyanate
JNK	c-Jun NH2-terminal kinase
MAPK	mitogen-activated protein kinase
MTT	3-(4,5-dimethylthiazol-2-ly)-2,5-diphenyl tetrazolium bromide
PARP	poly(ADP-ribose) polymerase
PBS	phosphate-buffered saline
PI	propidium iodide
ROS	reactive oxygen species
